# Astrocytic Redox Homeostasis as a Metabolic Modulator of DNA Damage and Repair in the Ischemic Penumbra

**DOI:** 10.3390/cells15121103

**Published:** 2026-06-18

**Authors:** Renata Kołodziejska, Antoni Godlewski, Agnieszka Tafelska-Kaczmarek, Julia Kuk, Magdalena Moritz, Krzysztof Sergot, Natalia Kurhaluk, Halina Tkaczenko, Alina Woźniak

**Affiliations:** 1 Department of Medical Biology and Biochemistry, Faculty of Medicine, Collegium Medicum in Bydgoszcz, Nicolaus Copernicus University in Toruń, Karłowicza 24, 85-092 Bydgoszcz, Poland; 2 Student Scientific Club of Biochemistry and Bioorganic Chemistry, Department of Medical Biology and Biochemistry, Faculty of Medicine, Collegium Medicum in Bydgoszcz, Nicolaus Copernicus University in Toruń, Karłowicza 24, 85-092 Bydgoszcz, Poland; 319905@stud.umk.pl (A.G.); 319910@stud.umk.pl (J.K.); 319915@stud.umk.pl (M.M.); 3 Department of Organic Chemistry, Faculty of Chemistry, Nicolaus Copernicus University in Toruń, Gagarina 7, 87-100 Toruń, Poland; tafel@umk.pl; 4 Institute of Applied Radiation Chemistry, Laboratory of Laser Molecular Spectroscopy, Faculty of Chemistry, Lodz University of Technology, Wroblewskiego 15, 93-590 Lodz, Poland; krzysztofsergot@gmail.com; 5 Department of Animal Physiology, Institute of Biology, Pomeranian University in Słupsk, Arciszewskiego 22a, 76-200 Słupsk, Poland; natalia.kurhaluk@upsl.edu.pl (N.K.); halina.tkaczenko@upsl.edu.pl (H.T.)

**Keywords:** ischemic stroke, astrocytes, ROS/RNS, DNA damage, therapeutic strategies

## Abstract

**Highlights:**

**What are the main findings?**

- Astrocytes regulate neuronal resistance to oxidative DNA damage in ischemic penumbra by maintaining redox balance, NAD^+^ levels, and metabolic support.
- PARP1-driven NAD^+^ depletion links DNA damage to bioenergetic failure, positioning metabolism as a key determinant of DNA repair efficiency after stroke.

**What are the implications of the main findings?**

- Targeting astrocyte-mediated metabolic and redox pathways may enhance DNA repair capacity and improve neuronal survival after ischemic stroke.
- Modulation of the redox–metabolic axis represents a promising therapeutic strategy to stabilize penumbral tissue and limit stroke-induced neurodegeneration.

**Abstract:**

Ischemic stroke triggers a severe redox disequilibrium that critically shapes cell survival within the penumbra. Although oxidative DNA damage arises from excessive ROS production, the capacity to repair such lesions is tightly constrained by cellular metabolic status. Growing evidence indicates that astrocytes, key metabolic regulators of the neurovascular unit, modulate neuronal susceptibility to genomic injury through redox buffering, NAD^+^ maintenance, and metabolic support. In the metabolically impaired yet structurally preserved penumbra, astrocytic control of glutathione turnover, mitochondrial function, and lactate shuttling may determine whether oxidative DNA lesions are efficiently repaired or progress toward energetic collapse. Poly(ADP-ribose) polymerase 1 activation following DNA strand breaks couples genomic stress to NAD^+^ depletion and bioenergetic failure, forming a critical interface between redox biology and metabolism. This framework posits that astrocytes preserve genomic integrity not by directly altering DNA repair pathways but by sustaining the energetic capacity required for an effective DNA damage response. Elucidating this astrocyte-centered redox–metabolic axis may reveal therapeutic strategies to stabilize penumbral tissue and improve stroke outcomes.

## 1. Introduction

Ischemic stroke remains a leading cause of mortality and long-term disability worldwide, and its pathophysiology extends far beyond the initial reduction in cerebral blood flow. Particular attention has been directed toward the ischemic penumbra, a dynamic region of structurally preserved yet metabolically compromised tissue, in which cellular fate is determined by a delicate balance between injury and adaptive responses. Within this region, disturbances in redox homeostasis emerge as critical determinants of neuronal survival, influencing both genomic integrity and recovery capacity [1].

Excessive production of reactive oxygen species (ROS), resulting from mitochondrial dysfunction and disruption of the electron transport chain, leads to the accumulation of oxidative DNA damage, including strand breaks and modifications of nitrogenous bases. However, the mere presence of genomic lesions does not necessarily determine cell death; rather, the critical factor is the cell’s capacity for efficient DNA repair, which is tightly dependent on the availability of energetic resources and overall metabolic balance. Under ischemic conditions, the limited availability of nicotinamide adenine dinucleotide (NAD^+^) and adenosine triphosphate (ATP) significantly impairs DNA repair pathways, such as base excision repair (BER), leading to the accumulation of damage and progression toward cellular demise [2,3].

Emerging evidence highlights astrocytes as central regulators of the redox–metabolic axis within the neurovascular unit. Through their capacity to buffer oxidative stress, regulate glutathione turnover, and provide metabolic substrates to neurons, particularly via lactate shuttling, astrocytes critically influence neuronal resilience in the penumbra. Moreover, astrocytes play a pivotal role in maintaining intracellular NAD^+^ pools and supporting mitochondrial function, thereby preserving the energetic capacity required for adaptive stress responses [4,5,6].

A key molecular interface linking genomic stress to metabolic failure is the activation of Poly(ADP-ribose) polymerase 1. Upon sensing DNA strand breaks, PARP1 catalyzes the transfer of ADP-ribose units from NAD^+^ onto target proteins, thereby initiating poly(ADP-ribosyl)ation as part of the cellular DNA damage response. While this process is essential for DNA repair signaling, excessive activation under severe oxidative stress leads to rapid NAD^+^ depletion and subsequent bioenergetic collapse. Thus, PARP1 functions as a critical switch between adaptive repair and cell death, integrating redox imbalance with metabolic dysfunction [7,8].

In this context, we propose that astrocytes preserve neuronal genomic integrity not by directly modulating the DNA repair machinery, but by sustaining the metabolic and energetic environment necessary for effective DNA damage responses. This astrocyte-centered redox–metabolic axis may represent a crucial determinant of tissue viability within the ischemic penumbra. Elucidating these mechanisms could uncover novel therapeutic strategies aimed at stabilizing vulnerable brain regions and improving outcomes following ischemic stroke [9,10,11].

## 2. The Ischemic Penumbra as a Redox–Metabolic Microenvironment

The ischemic penumbra represents a highly dynamic and heterogeneous microenvironment characterized by metabolic instability and tightly regulated transcriptional responses. It is defined as hypoperfused but potentially salvageable tissue, in which residual energy metabolism is preserved despite reduced oxygen and glucose availability [12,13]. Importantly, the penumbra is not a static entity but rather a temporally and spatially evolving region, in which cellular fate is determined by thresholds of hypoxia, the duration of ischemia, and the efficiency of reperfusion [13]. This environment is associated with extensive transcriptional remodeling, including activation of stress-response genes and cell-specific molecular programs that regulate survival or progression toward cell death [13,14]. A key component of this adaptive response is the activation of hypoxia-inducible pathways. Hypoxia-inducible factor 1α (HIF-1α) acts as a master regulator of cellular adaptation to reduced oxygen availability, promoting angiogenesis, metabolic reprogramming, and cell survival [15]. HIF signaling induces the expression of genes involved in glycolysis, glucose transport, and oxygen homeostasis, enabling cells to maintain ATP production under hypoxic conditions [15,16]. This shift from oxidative phosphorylation to glycolysis represents a fundamental metabolic adaptation within the penumbra, allowing temporary preservation of bioenergetic balance despite mitochondrial dysfunction [16].

Reactive oxygen species play a central role in this environment as both damaging agents and signaling molecules. At physiological levels, ROS regulate intracellular signaling pathways by reversible redox modifications of proteins and by activating transcription factors such as nuclear factor kappa-light-chain-enhancer of activated B cells (NF-κB), nuclear factor erythroid 2-related factor 2 (Nrf2), and HIF-1α [17]. However, during ischemia and reperfusion, excessive ROS production leads to oxidative stress and cellular injury. Mitochondrial dysfunction, NADPH oxidase activation, and other enzymatic sources contribute to increased ROS generation, particularly during reperfusion when oxygen is rapidly reintroduced [14,18]. This results in oxidative damage to lipids, proteins, and nucleic acids, contributing to neuronal death.

Reactive nitrogen species (RNS), particularly nitric oxide (NO) and its derivatives, further amplify redox imbalance. Ischemia induces activation of different nitric oxide synthase (NOS) isoforms, including neuronal (nNOS), endothelial (eNOS), and inducible (iNOS), leading to increased NO production [19]. While eNOS-derived NO may support perfusion through vasodilation, excessive NO production from nNOS and iNOS contributes to neurotoxicity [19]. Importantly, NO reacts with superoxide to form peroxynitrite, a highly reactive species that induces lipid peroxidation, protein nitration, and DNA damage [18,19].

A critical downstream consequence of oxidative and nitrosative stress is DNA damage and activation of repair pathways [19]. This mechanism highlights the direct link between redox imbalance and metabolic collapse within the penumbra.

The penumbra also represents a key interface between oxidative stress and neuroinflammation. ROS act as upstream signaling molecules that activate inflammatory pathways, while inflammatory cells further enhance ROS and RNS production, creating a self-amplifying cycle [14,18]. Microglia are central players in this process. Upon activation, microglia upregulate iNOS and produce large amounts of NO, contributing to nitrosative stress and increasing neuronal susceptibility to DNA damage [18,19]. In addition, microglia-derived ROS and cytokines amplify local oxidative stress and influence neuronal survival. Pro-inflammatory cytokines such as tumor necrosis factor-alpha (TNF-α) and interleukin-1 beta (IL-1β) further modulate the redox-metabolic environment. These mediators regulate intracellular signaling pathways that influence ROS production, mitochondrial function, and antioxidant defenses [12,14]. Their effects are highly context-dependent: while excessive activation promotes injury, controlled signaling may contribute to adaptive responses, including induction of antioxidant systems and stress–response pathways. This highlights the dual role of inflammation in shaping redox homeostasis within the penumbra.

In summary, the ischemic penumbra can be conceptualized as a redox-metabolic microenvironment in which hypoxia-driven transcriptional regulation, metabolic reprogramming, oxidative stress, and neuroinflammation are tightly interconnected. The balance between these processes determines whether cells adapt or undergo irreversible damage. Astrocytes contribute significantly to determining the fate of the penumbra by modulating metabolic homeostasis and the redox environment. Through increased glutamate uptake, they help reduce excitotoxicity; by providing neurons with metabolic substrates, they support energy metabolism under ischemic conditions; and by regulating antioxidant systems, they enhance cellular resilience to oxidative stress. In addition, astrocytes participate in shaping the inflammatory response through cytokine release and interactions with microglia, thereby influencing the balance between neuroprotective and potentially detrimental processes [9,20].

## 3. Sources of Oxidative Stress and Its Impact on DNA

Ischemia triggers complex pathophysiological cascades in which reactive oxygen species play a key role. Under physiological conditions, ROS act as signaling molecules, modulating protein activity through reversible redox modifications and regulating synaptic plasticity. In a stroke, the most important species are hydrogen peroxide and the hydroxyl radical [21]. Hydrogen peroxide at low concentrations regulates transcription factors such as Nrf2 and HIF-1α, as well as kinase signaling pathways, whereas the highly reactive hydroxyl radical causes non-selective damage to macromolecules [17,22,23].

Mitochondria are the main source of ROS during ischemia. Disruption of the Krebs cycle and oxidative phosphorylation leads to succinate accumulation, while oxygen deprivation inhibits electron transport, causing electron leakage from complexes I and III and a shift toward anaerobic glycolysis [14,22,23].

Reperfusion leads to rapid oxidation of accumulated succinate and a massive influx of electrons into the respiratory chain. Exceeding its electron transfer capacity initiates reverse electron transport (RET), in which electrons flow back to complex I and are directly transferred to oxygen, generating a characteristic mitochondrial ROS “burst” [14,22,23].

Another major source of ROS is NADPH oxidases (NOX), which transfer electrons from NADPH to oxygen, producing superoxide anion. Although NOX activity normally serves signaling functions, it is markedly increased after stroke. Hypoxia upregulates NOX4 in neurons and NOX2 in microglia, whose pro-inflammatory activation further enhances ROS production and neuronal injury [14,22,23,24,25].

The generated superoxide anion is converted by superoxide dismutase (SOD) into hydrogen peroxide. At the same time, ionic imbalance and acidosis promote the release of transition metal ions (Fe^2+^, Cu^+^), which catalyze Fenton and Haber–Weiss reactions, generating highly reactive hydroxyl radicals, major mediators of oxidative damage [14,22,23]. Although initiated by different mechanisms, mitochondrial and NOX-derived ROS converge on common pathways that lead to irreversible cell death. Under oxidative stress, guanine is particularly vulnerable to oxidation because it has the lowest oxidation potential among nucleobases [22,26].

**Figure 1:**
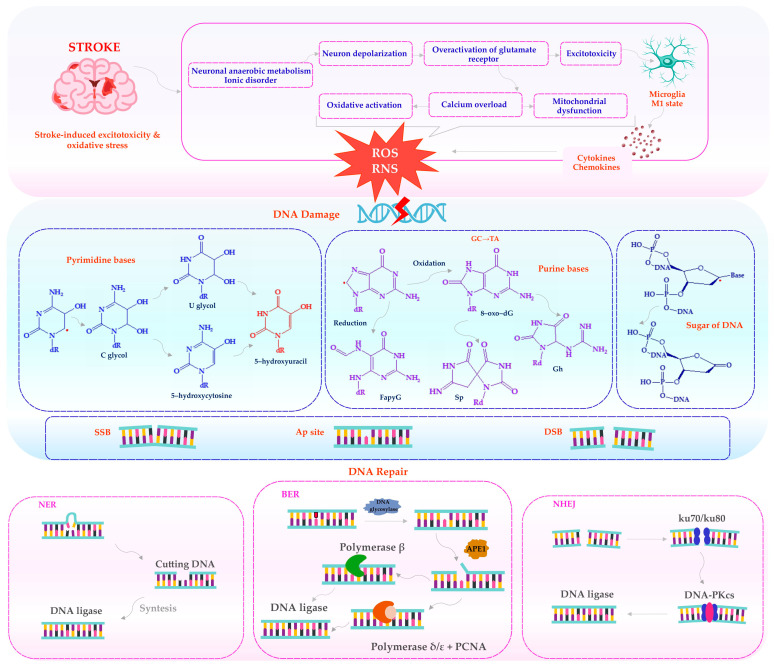
Oxidative DNA base lesions and their consequences in cerebral ischemia. Different types of oxidative DNA damage occur under ischemia–reperfusion conditions. Hydroxyl radicals generate 8-oxoguanine, its further oxidation products, formamidopyrimidines, modified pyrimidines, and AP sites. The accumulation of these lesions leads to the formation of single- and double-stranded breaks, transcriptional disturbances, PARP1 activation, and overload of the BER, NER, and NHEJ pathways, which promotes neuronal degeneration in the ischemic penumbra. Abbreviations: APE1—apurinic/apyrimidinic endonuclease 1; Ap—apurinic/apyrimidinic site; BER—base excision repair; C—cytosine; dG—deoxyguanosine; DNA—deoxyribonucleic acid; DSB—double-strand break; FapyG—formamidopyrimidine-guanine; GC—guanine–cytosine pair; Gh—guanidinohydantoin; NHEJ—non-homologous end joining; NER—nucleotide excision repair; PCNA—proliferating cell nuclear antigen; DNA–PKcs-dependent protein kinase catalytic subunit; RNS—reactive nitrogen species; ROS–reactive oxygen species; SSB—single-strand break; Sp—spiroiminodihydantoin; TA—thymine–adenine pair; U—uracil.

The most common product of guanine oxidation is 8-oxoguanine (8-oxoG). It is formed as a result of hydroxyl radical attack at the C8 position of the purine ring (Figure 1). 8-oxoG has a strong mutagenic potential. During replication, DNA polymerase frequently mispairs it with adenine, leading to GC → TA transversions [27,28]. Further oxidation produces lesions such as guanidinohydantoin (Gh) and spiroiminodihydantoin (Sp), which inhibit RNA polymerase II elongation, impair protein synthesis, and are poorly recognized by 8-oxoguanine DNA glycosylase 1 (OGG1). Consequently, DNA damage accumulates, and cell death pathways are activated. In the ischemic penumbra, limited energy availability further compromises repair mechanisms and accelerates neuronal degeneration [14,25,27].

Oxidative stress can also cause extensive purine degradation. Under hypoxic and acidic conditions, guanine radical adducts undergo reduction and ring opening, forming FapyG and FapyA. These lesions are inefficiently repaired by base excision repair and are associated with worse stroke outcomes and increased apoptotic signaling [25,27,29,30].

Hydroxyl radicals also damage pyrimidines by attacking the C5=C6 bond, generating thymine glycol, uracil glycol, dihydrothymine, and 5-hydroxycytosine, which may be deaminated to mutagenic 5-hydroxyuracil [22,28,30]. These lesions are removed by NEIL, a DNA glycosylase (NEIL), and nei-like DNA glycosylase 3 (NEIL3), which plays a key role in progenitor cells and neurogenesis [25,27].

Accumulation of oxidized bases promotes higher-order DNA damage, including single-strand breaks caused by direct hydroxyl radical attack on deoxyribose and by secondary radicals generated during lipid peroxidation and protein oxidation [25,31].

Double-strand breaks (DSBs) are the most destructive form of genomic damage and arise during ischemia–reperfusion through both direct and enzymatic mechanisms. In the direct mechanism, DSBs occur when two independent oxidative lesions arise in close proximity on opposite strands, leading to loss of helix continuity [25,28].

Hydroxyl radicals can also induce abasic apurinic/apyrimidinic (AP) sites by attacking deoxyribose, generating sugar radicals, and cleaving the N-glycosidic bond. AP sites are particularly increased during reperfusion, and their subsequent oxidation further increases the DNA backbone’s susceptibility to additional damage [27,28,31].

Cells respond to oxidative DNA damage by activating non-homologous end joining (NHEJ), nucleotide excision repair (NER), and base excision repair (BER), the latter being the principal pathway for repairing oxidative lesions after ischemic stroke [28,31]. BER is initiated by DNA glycosylases, followed by AP-site cleavage by apurinic/apyrimidinic endonuclease 1 (APE1). Repair proceeds through short-patch (DNA polymerase β) or long-patch (DNA polymerases δ/ε) synthesis and is completed by DNA ligases [31]. In ischemia, impaired energy supply disrupts BER efficiency, leading to toxic repair intermediates and strand breaks, while excessive PARP1 activation further depletes NAD^+^ and ATP, aggravating neuronal injury [31,32].

In postmitotic neurons, the dominant pathway for repairing double-strand breaks is NHEJ, initiated by the Ku70/Ku80 protein complex and supported by histone H2AX (H2A histone family member X) phosphorylation and recruitment of p53-binding protein 1 (53BP1) [31]. This mechanism is rapid but error-prone, making it efficient yet imprecise [25,32]. NER removes lesions that strongly distort the DNA helix, and its efficiency depends, among others, on excision repair cross-complementation group 1 (ERCC1), whose expression increases in the ischemic penumbra and supports neuronal survival [24,27,31].

Reactive nitrogen species (RNS) further contribute to ischemic injury. Activated microglia in the penumbra produce nitric oxide (NO) and peroxynitrite (ONOO^−^), exacerbating mitochondrial dysfunction and energy deficits [26,33]. RNS induce DNA base modifications and strand breaks, activating energy-consuming repair processes that, under ATP-depleted conditions, accelerate the transition from reversible dysfunction to irreversible neuronal death [22,33].

Nitrosative products also destabilize the blood–brain barrier and amplify local inflammation. The extent of damage depends partly on the microglial phenotype: a predominance of the pro-inflammatory phenotype increases RNS production, whereas a reparative phenotype may limit tissue injury [23].

Overall, oxidative and nitrosative stress are central mediators of ischemic brain injury. Excessive ROS and RNS production causes extensive DNA damage, while impaired repair mechanisms resulting from energy depletion lead to the accumulation of genomic lesions, neuronal death, and progression of ischemic damage.

## 4. Astrocytes as a Regulator of the Redox Environment

Astrocytes form a functional syncytium, and neurons exert a profound influence on their phenotype [34,35]. This involves the activation of hundreds of genes and the repression of hundreds of others; astrocytes achieve a fully mature phenotype only in the presence of neurons [36]. Neuronal signals also play a key role in enhancing astrocytic antioxidant defenses through multiple pathways [37,38]. Astrocytes exhibit significantly higher expression of antioxidant enzymes, such as mitochondrial superoxide dismutase, heme oxygenase-1 (HO-1), and glutathione peroxidase (GPx), than neurons, and these enzymes are regulated by the Nrf2 signaling pathway in response to oxidative stress [39,40].

**Figure 2:**
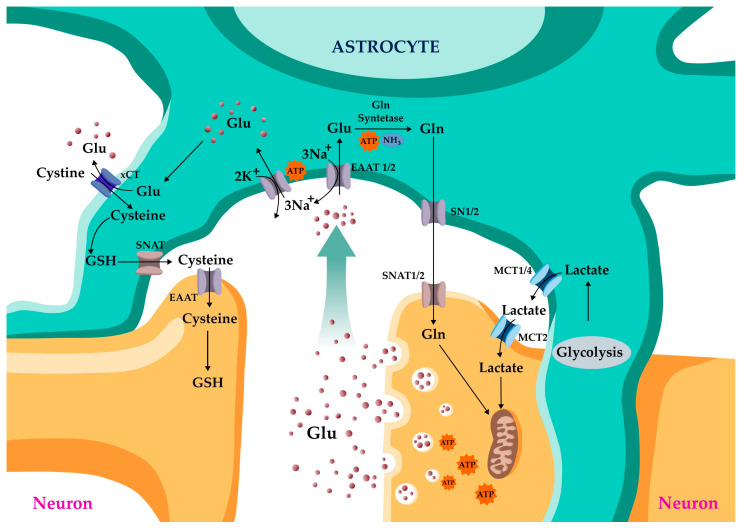
Schematic overview of selected pathways involved in molecular exchange between astrocytes and neurons. Glutamate uptake from the synaptic cleft is mediated by excitatory amino acid transporters 1 and 2 (EAAT1/2) and depends on the transmembrane sodium gradient maintained by the sodium–potassium adenosine triphosphatase (Na^+^/K^+^-ATPase). Within astrocytes, glutamate is converted into glutamine by adenosine triphosphate-dependent glutamine synthetase (GS), after which glutamine is transported back to neurons. Astrocytic glycolysis generates lactate, which is exported via monocarboxylate transporters 1 and 4 (MCT1/4) and imported into neurons through monocarboxylate transporter 2 (MCT2), where it supports oxidative metabolism. Astrocytes also take up cystine from the extracellular space, reduce it to cysteine, and support neuronal glutathione synthesis by providing substrates. Abbreviations: ATP—adenosine triphosphate; EAAT—excitatory amino acid transporter; Gln—glutamine; Glu—glutamate; GSH—glutathione; MCT1/4—monocarboxylate transporter 1/4; MCT2—monocarboxylate transporter 2; SN1/2—sodium-coupled neutral amino acid transporter 1/2; SNAT—sodium-coupled neutral amino acid transporter; xCT—cystine/glutamate antiporter.

Neurons and astrocytes exhibit distinct metabolic profiles. In the presence of oxygen, neurons metabolize glucose primarily via oxidative pathways, whereas glucose taken up by astrocytes is preferentially processed through glycolysis (Figure 2) [41,42]. The resulting lactate is released and taken up by neurons, which efficiently use it as a substrate for oxidative metabolism [43,44]. In addition to its energetic role, lactate may reduce mitochondrial ROS production by stabilizing the NADH/NAD^+^ ratio and alleviating electron transport chain overload [45]. This phenomenon is referred to as the astrocyte–neuron lactate shuttle (ANLS). Lactate is exported to the extracellular space via monocarboxylate transporters (MCTs), from where it is transported into neurons via neuronal MCTs [46]. This process is driven, among other factors, by a concentration gradient—astrocytes contain higher lactate levels than neurons [43]. Disruption of ANLS under ischemic conditions leads to increased mitochondrial burden in neurons, elevated ROS production, and a higher risk of DNA damage [47]. However, this model remains a subject of ongoing debate.

Astrocytes preferentially express lactate dehydrogenase 5 (LDH5, composed of LDHA subunits), whereas neurons predominantly express lactate dehydrogenase 1 (LDH1, composed of LDHB subunits). Both isoforms catalyze the same reversible reaction and can operate in either direction. However, they differ in their kinetic properties and thus in their preferred directionality. LDH1 (neuronal) favors the conversion of lactate to pyruvate, consistent with lactate utilization in oxidative phosphorylation. In contrast, LDH5 (astrocytic) favors the conversion of pyruvate to lactate, supporting high glycolytic flux and NAD^+^ regeneration [48,49]. While their kinetic properties in vitro suggest that LDH1 favors lactate utilization and LDH5 supports production [47,48,49], the actual in vivo directionality depends heavily on local substrate/product ratios and cellular workloads. Critics of the ANLS model, such as Dienel, argue that during intense neuronal activation, neurons accelerate their own glycolysis and can produce or accumulate lactate rather than strictly importing it [50]. Thus, cellular expression profiles alone do not definitively prove net lactate flux from astrocytes to neurons.

Neurons utilize glucose sparingly for glycolysis, channeling a significant portion into the pentose phosphate pathway (PPP). This is enabled in part by low expression of 6-phosphofructo-2-kinase/fructose-2,6-biphosphatase 3 (PFKFB3), an enzyme that generates fructose-2,6-bisphosphate, a potent allosteric activator of phosphofructokinase-1 [51]. However, alternative models emphasize that this metabolic allocation is highly dynamic. Under conditions of high workload or oxidative stress, neurons can upregulate glycolysis and directly utilize glucose to meet their energetic demands, challenging the view that they are strictly dependent on astrocytic lactate [50]. In astrocytes, mitochondrial oxidation of pyruvate is functionally associated with high expression of pyruvate dehydrogenase kinase 4 (PDK4), which phosphorylates and inhibits the pyruvate dehydrogenase complex (PDH) [52]. In astrocytes, high PPP activity serves as a major source of NADPH, which is essential for the regeneration of reduced glutathione (GSH) and thus directly determines the capacity to neutralize ROS [53]. Additionally, one-carbon metabolism (folate and methionine cycles) supports NADPH production and integrates redox control with cellular metabolism [54].

Glycolysis and glycogenolysis are essential for the proper functioning of astrocytes because their tiny cellular processes are extremely narrow and cannot accommodate many mitochondria. Consequently, cytoplasmic ATP production and diffusion of ATP and phosphocreatine play a critical role [55,56]. Astrocytic glycogen is also crucial for neuronal activity [57]; for example, inhibition of astrocytic glycogenolysis can impair memory consolidation [58]. Similarly, the lactate shuttle is essential for long-term memory formation and maintenance of long-term potentiation [59]. Interestingly, excessive lactate supply may increase pain sensitivity and contribute to neuropathic pain pathophysiology [60].

Neurons incorporate most of complex I into respiratory chain supercomplexes, promoting efficient electron transfer and minimizing electron leakage to oxygen. Astrocytes, in contrast, possess a larger pool of “free” complex I, which is more prone to electron leak. This feature may support their elevated antioxidant capacity [61,62]. Astrocytes may also stabilize neuronal mitochondrial membrane potential, limiting the propagation of oxidative stress [63]. Astrocytic peroxisomes contribute to ROS detoxification via catalase and other oxidases, providing an additional layer of redox control [64]. Nevertheless, mitochondrial ROS contribute to the development of dementia [65].

Maintenance of redox balance is essential for cell survival. For example, during mitochondrial outer membrane permeabilization and cytochrome c release, only the oxidized form (Fe^3+^) of cytochrome c effectively binds Apaf-1 and initiates caspase-9 activation [66]. Astrocytes are significantly better equipped than neurons for antioxidant defense. Their pentose phosphate pathway activity is five- to sevenfold higher [67]. Astrocytes also serve as the primary source of glutathione precursors for neurons, which have limited capacity for its synthesis, making them dependent on astrocytic GSH metabolism [68]. Their proteome is broadly adapted to maintain homeostasis under stress conditions. A key regulator is Nrf2, which is nearly absent in neurons due to constitutive degradation mediated by kelch-like ECH-associated protein 1 (Keap1) [69,70]. Nrf2 activates ARE (antioxidant response element)-dependent genes, including those essential for glutathione synthesis [71]. Its presence protects neurons in mixed (astrocyte-containing) primary cultures [72].

Nrf2 activation in astrocytes is promoted, among others, by glutamatergic signaling via N-methyl-D-aspartate (NMDA) receptors. In astrocytes, glutamate signaling functions as an anticipatory cue for increased neuronal activity and impending redox burden. Therefore, astrocytes activate Nrf2 primarily through receptor-mediated pathways, rather than waiting for ROS accumulation [73]. An additional relevant interaction is that, under inflammatory conditions, microglia-derived nitric oxide likely modifies Keap1 (via cysteine S-nitrosylation), leading to Nrf2 release [74]. Activated microglia also serve as a source of ROS, further stimulating astrocytic antioxidant responses [75].

In astrocytes, Nrf2 activation strongly induces expression of the SLC7A11 transporter (system xCT), increasing cystine uptake from the extracellular space [76]. Cystine is reduced to cysteine at the expense of NADPH and used for intensive glutathione synthesis or exported back into the extracellular space to supply neurons. Neurons, due to low system xCT activity and limited ability to directly uptake cystine or intact glutathione, are functionally dependent on this pathway for reduced cysteine supply, which is rate-limiting for their glutathione synthesis. This cycle is critically important; co-culture with astrocytes prevents neuronal death and damage induced by mitochondrial pro-oxidant toxins [71,77]. In general, exposure to severe, potentially lethal stress may sensitize neurons to subsequent insults, exacerbating toxicity [78,79]. In contrast, mild sublethal stress can induce tolerance and protect against subsequent insults [80]. Astrocytes that survive strong oxidative stress exhibit resistance to further oxidative challenges and display elevated Nrf2 and glutathione levels. However, glutathione is not the sole determinant of oxidative stress resistance—Nrf2 engages multiple protective mechanisms [81]. Astrocytes also show high resistance to proteotoxic stress induced by proteasome inhibition, maintaining adaptive capacity (“second hit protection”). Despite the accumulation of misfolded proteins, glutathione depletion does not occur, indicating highly efficient redox control, although glutathione remains essential for cell survival [82].

The cystine–glutamate antiporter (system xCT) can be activated by Nrf2, increasing cystine uptake and glutathione synthesis. However, this is coupled with glutamate export into the extracellular space [83]. This mechanism reflects a trade-off between antioxidant protection and potential exacerbation of excitotoxicity [84]. Astrocytes can modulate NMDA receptor activation threshold via the co-agonist D-serine, whose synthesis is reduced under severe oxidative stress due to increased glutathione production [85]. It is noteworthy that astrocytes may also release glutamate into the synaptic space in response to inflammatory signals [86,87].

One of the most important roles of astrocytes is the removal of glutamate from the synaptic cleft. Subsequently, in an ATP-dependent reaction, glutamine synthetase converts glutamate and ammonium into glutamine, also contributing to ammonia detoxification. This prevents excessive receptor stimulation and protects neurons from excitotoxicity. The resulting glutamine is transported back to neurons, where it is converted again into glutamate and replenishes synaptic vesicles [88,89].

Glutamate uptake is energetically demanding and occurs via co-transport with three sodium ions. The required sodium gradient is maintained by Na^+^/K^+^-ATPase, resulting in an effective cost of one ATP molecule per glutamate transported [90,91,92]. An additional ATP molecule is consumed during its conversion to glutamine [93].

Under physiological conditions, the rate-limiting factor for glutamate uptake appears to be transporter abundance rather than energy availability [94]. In astrocytes, glutamate can enter the tricarboxylic acid (TCA) cycle for energy production, although this is not its primary fate [95]. Astrocytes support neuronal TCA cycle function via glutamine supply, as neurons lack the capacity to replenish oxaloacetate from pyruvate [96].

Astrocytes do not avoid oxidative phosphorylation [97]. To understand energy dynamics, it is important to note that phosphocreatine diffuses much faster than adenine nucleotides and shuttles energy to sites of high demand. As a result, ADP does not need to diffuse back over long distances to mitochondria. This system acts as both an energy buffer and a transport mechanism, maintaining stable ATP, ADP, and inorganic phosphate levels despite fluctuations in demand [98,99]. ADP stimulates mitochondrial enzymes as a substrate in direct response to ATP consumption [100], whereas anticipatory regulation is mediated by calcium ions. Intracellular Ca^2+^ increases in response to glutamate via several mechanisms, primarily through activation of metabotropic glutamate receptors on astrocytes, leading to Ca^2+^ release from the endoplasmic reticulum [101,102]. Increased cytosolic Na^+^ due to glutamate co-transport can activate the Na^+^/Ca^2+^ exchanger (NCX), which under these conditions operates in reverse mode, importing Ca^2+^ into the cell. Elevated Ca^2+^ enhances glucose transport via glucose transporter type 1 (GLUT1) [103] and activates, among others, the malate–aspartate shuttle (MAS), increasing NADH delivery to mitochondria [104], as well as key oxidative enzymes such as isocitrate dehydrogenase and α-ketoglutarate dehydrogenase [105,106,107,108,109,110].

Reactive astrocytes are a phenotype that emerges in response to injury. This involves hypertrophy and extensive transcriptomic reprogramming. Their primary role is stabilization of the neuronal microenvironment under stress conditions: they enhance glutamate uptake, buffer potassium ions, and secrete various signaling molecules, including gamma-aminobutyric acid (GABA), ATP, and cytokines. In Alzheimer’s disease, astrocyte-derived GABA contributes to neurodegeneration. Reactive astrocytes also form a physical and functional barrier around injury sites, limiting the spread of inflammation and toxins, but chronically inhibiting axonal regeneration and plasticity [111,112]. Under chronic stress conditions, astrocytes may enter a senescent state characterized by the senescence-associated secretory phenotype (SASP), involving the release of pro-inflammatory cytokines and ROS, thereby exacerbating oxidative stress and DNA damage in neurons [113].

Under normoxic conditions, prolyl hydroxylase domain enzymes (PHDs) use molecular oxygen to hydroxylate HIF-1α, targeting it for ubiquitination and proteasomal degradation. PHDs require oxygen, Fe^2+^, and 2-oxoglutarate as cofactors. Consequently, not only hypoxia but also increased ROS, resulting from electron accumulation in the respiratory chain due to limited availability of terminal electron acceptors, reduces their activity [114,115]. There is a tight crosstalk between hypoxia-inducible factor 1, ROS, and Nrf2, in which ROS can stabilize HIF-1α and activate Nrf2, forming an integrated adaptive response system in the penumbra [116,117]. Additionally, accumulation of TCA cycle metabolites such as succinate and fumarate inhibits PHDs by competing with α-ketoglutarate [118,119,120]. High HIF-1α levels are characteristic of stem cells and cancer cells; in these contexts, increased levels result not only from stabilization but also from enhanced translation driven by mechanistic target of rapamycin complex 1 (mTORC1) activation [121,122]. One of the key downstream effectors of HIF-1α is pyruvate dehydrogenase kinase 1 (PDK1), which phosphorylates and inhibits PDH, thereby limiting electron flux into the respiratory chain [119,123].

Even conditions such as sleep apnea can induce detrimental changes in the hippocampus, including inflammation and significant epigenetic alterations. The DNA demethylase TET1 (ten-eleven translocation 1) acts as a transcriptional coactivator of HIF-1, and chronic activation of HIF target genes may be both pro-inflammatory and oncogenic [124]. HIF-1 plays an important role in shaping immune responses to hypoxia [125], and its activation constitutes an effective adaptive response to low oxygen conditions, with overall protective effects [126].

Overall, astrocytes are important modulators of neuronal responses to metabolic and oxidative stress. By regulating redox balance, energy substrate availability, and glutamate homeostasis, they contribute to cellular adaptation and may help limit mechanisms associated with oxidative damage. These diverse functions place astrocytes at the interface of metabolic regulation, antioxidant defense, and neuroprotection.

## 5. Astrocytic Modulation of DNA Damage and Repair in Neurons

During ischemic stroke, neurons rapidly accumulate DNA damage caused by oxidative stress, energy depletion, and mitochondrial dysfunction. As endogenous repair systems become insufficient, cells transition from reversible dysfunction to irreversible death. Simultaneously, ROS levels rise markedly. While ROS normally serve signaling functions, during ischemia they induce DNA base modifications, abasic sites, and strand breaks [127,128,129].

These lesions activate the DNA damage response (DDR), in which PARP1 plays a central role. This enzyme catalyzes poly(ADP-ribose) synthesis, recruits BER-associated proteins such as XRCC1 (X-ray repair cross-complementing protein 1) and DNA polymerase β, and supports the repair of oxidative DNA damage [130,131,132]. The nucleotide excision repair pathway complements BER by removing lesions that significantly distort the DNA helix [133]. However, excessive oxidative stress leads to PARP1 overactivation, resulting in NAD^+^ and ATP depletion, mitochondrial dysfunction, and secondary ROS generation, thereby amplifying cellular damage [132,134].

Independent of astrocyte-mediated support, neurons rely primarily on their intrinsic DNA repair machinery to maintain genomic integrity. Among the available pathways, base excision repair is the predominant mechanism for removing oxidative DNA lesions generated during ischemia, including oxidized bases, abasic sites, and single-strand breaks. Key BER proteins such as OGG1, APE1, XRCC1, and DNA polymerase β play essential roles in maintaining neuronal genome stability. In addition, nucleotide excision repair contributes to the removal of bulky DNA lesions, whereas non-homologous end joining represents the major pathway for repairing double-strand breaks in post-mitotic neurons. Experimental studies suggest that impairment of these repair systems increases neuronal vulnerability to ischemic injury, highlighting the importance of neuron-intrinsic DNA repair mechanisms as a first line of defense against ischemia-induced genomic instability. Nevertheless, the efficiency of these pathways is strongly influenced by the cellular metabolic and redox environment, creating a potential interface through which astrocytes may indirectly modulate neuronal DNA repair capacity [28,135,136].

At this critical point, astrocytes assume a key protective role. As one of the first cell types to respond to ischemia, they undergo metabolic and secretory reprogramming. Astrocytes release trophic, antioxidant, and anti-inflammatory factors that reduce oxidative stress in neurons and may indirectly support DNA repair processes by preserving cellular homeostasis. They also stabilize the penumbral microenvironment by regulating energy substrate availability and modulating neuroinflammation, both of which could influence the efficiency of BER, NER, and NHEJ pathways in neurons. Consequently, astrocytes may represent important modulators of the neuronal response to DNA damage by shaping the metabolic, redox, and inflammatory microenvironment. However, direct evidence demonstrating that astrocytes regulate specific neuronal DNA repair pathways in the ischemic penumbra remains limited, and this concept requires further experimental validation [137].

Persistent PARP1 overactivation may trigger parthanatos, a caspase-independent form of cell death associated with excessive poly(ADP-ribose) accumulation. A key event is the release of apoptosis-inducing factor (AIF) from mitochondria and its translocation to the nucleus, where it promotes extensive DNA fragmentation and chromatin condensation. This mechanism contributes significantly to neuronal injury in stroke and neurodegenerative disorders [131,134,138]. In the context of this ROS-induced damage cascade, astrocytes play a key protective role, acting as a central component of redox and metabolic homeostasis in the neuronal microenvironment. Compared to neurons, these cells exhibit a more efficient antioxidant system and greater metabolic flexibility, allowing them to function as a buffer against oxidative stress [139,140]. A major protective mechanism involves the glutamine pathway, through which astrocytes provide glutathione (GSH) precursors that neurons use to neutralize ROS and maintain redox balance [140]. Activation of the Nrf2 signaling pathway further enhances antioxidant capacity by inducing enzymes such as superoxide dismutase, catalase, and those involved in NADPH regeneration. Astrocytes also support neuronal energy metabolism by supplying lactate through the astrocyte–neuron lactate shuttle, thereby sustaining ATP production under conditions of limited oxygen availability [76,139,141]. Through these mechanisms, astrocytes effectively reduce ROS levels and stabilize neuronal metabolism, thereby decreasing the frequency and severity of DNA damage. Consequently, PARP enzyme activation is limited, and NAD^+^ consumption is reduced. Maintenance of adequate NAD^+^ levels ensures proper functioning of metabolic pathways, including glycolysis, the TCA cycle, and oxidative phosphorylation, preventing ATP depletion and mitochondrial dysfunction. At the same time, a stable redox environment and sufficient energy availability support the efficiency of fundamental DNA repair mechanisms such as BER and NER, which are essential for the removal of oxidative and structural DNA damage. In this way, astrocytes may indirectly facilitate neuronal DNA repair by maintaining favorable metabolic and redox conditions, potentially reducing the likelihood of PARP1 overactivation and subsequent neuronal death [142,143,144,145]. An additional layer of neuronal DNA damage regulation involves epigenetic mechanisms closely linked to cellular metabolism, energy status, and redox balance. Disturbances in one-carbon metabolism, including the folate and methionine cycles, affect the cellular methylation potential determined by the ratio of S-adenosylmethionine (SAM) to S-adenosylhomocysteine (SAH) [146,147,148]. Changes in methionine availability and one-carbon flux alter SAM levels, influencing DNA and histone methylation, chromatin organization, and accessibility to repair complexes [146]. Moreover, these changes may affect the activity of DNA damage response enzymes, such as PARP1, whose function is coupled to the cell’s metabolic state and cofactor availability [149]. Epigenetic modifications directly influence the expression of genes encoding proteins involved in major DNA repair pathways, including BER and NER, as well as in oxidative stress responses and redox homeostasis [146,150]. In neurons, this is particularly relevant in the context of transcription-associated DNA breaks, which may regulate immediate early gene expression and influence neuronal plasticity [151]. Under pathological conditions such as stroke, dysregulation of the SAM/SAH axis may lead to transcriptional disturbances that affect genes involved in genome stability, further impairing neurons’ ability to repair DNA damage [146,147]. Neurons are particularly sensitive to disruptions in metabolic homeostasis due to their high energy demands and limited regenerative capacity. Consequently, one-carbon metabolism represents a critical link between cellular metabolic state and epigenetic regulation of DNA damage responses, integrating metabolic signals with chromatin regulation and gene expression [146,152].

Although accumulating evidence supports this model, direct demonstration of astrocyte-mediated regulation of specific neuronal DNA repair pathways following ischemic stroke remains limited. Much of the available evidence derives from experimental models, in vitro studies, or related pathological conditions such as aging and neurodegenerative diseases. T Therefore, the proposed astrocyte–redox–DNA repair axis should currently be regarded as a conceptual framework supported by converging mechanistic evidence rather than a fully established pathway in human ischemic stroke. Further translational and clinical studies are needed to determine whether astrocytes directly modulate neuronal DNA repair and genomic stability after stroke [146,147,153].

In summary, ischemic stroke is associated with increased oxidative stress, DNA damage, and activation of cellular repair mechanisms. The efficiency of these responses may influence neuronal survival and functional recovery. Astrocytes contribute to maintaining redox and metabolic homeostasis, thereby supporting neuronal function and DNA repair processes. In addition, epigenetic mechanisms linked to one-carbon metabolism may modulate the expression of genes involved in genome maintenance and stress responses. Together, these interconnected pathways shape the cellular response to ischemic injury and may contribute to the overall outcome of stroke.

## 6. The Redox-Metabolic Threshold as a Transition Point to Irreversibility and Therapeutic Implications

Microvascular events occurring immediately after ischemic stroke initiate a cascade of processes that determine tissue fate. The early phase of ischemia is characterized by rapid energy failure, redox imbalance, and disruption of the neurovascular unit. These processes form a coupled, threshold-dependent metabolic system in which exceeding critical bioenergetic limits triggers an oxidative–mitochondrial cascade leading to cell death [154].

From a translational perspective, therapeutic strategies should focus on maintaining astrocytic redox–metabolic capacity to preserve DNA integrity within the penumbra. This includes sustaining NAD^+^ levels, controlling PARP1 activity, and integrating Nrf2-dependent antioxidant responses with PPP flux and GSH regeneration. Stabilization of mitochondrial function and preservation of astrocyte–neuron metabolic coupling are also essential, as they enable redistribution of energy substrates and buffering of oxidative stress. Additional targets include modulation of microglia–astrocyte interactions, support of one-carbon metabolism, and limitation of senescent cell accumulation through senolytic approaches or SASP modulation, thereby reducing chronic inflammation and secondary DNA damage [154,155,156].

These observations further emphasize the central role of astrocytes in maintaining neuronal metabolic stability and genomic integrity under ischemic conditions [146,147,153].

NAD^+^ is a central regulator of bioenergetics, redox balance, DNA repair, and mitochondrial homeostasis. In the ischemic penumbra, its levels rapidly decline, supporting therapeutic strategies aimed at maintaining NAD^+^ in astrocytes. This preserves mitochondrial stability, redox capacity, and DNA protection. Enhancing NAD^+^ pools extends the “reversibility window” of the penumbra and limits progression toward irreversible necrosis [154].

Beyond its role as a central regulator of bioenergetics and redox balance, NAD^+^ also serves as a cofactor for multiple enzymes, including sirtuin 1/3/6 (NAD^+^-dependent deacetylases), PARP1/2, CD38/CD157, and sterile alpha and TIR motif containing 1. It also plays a key role in DNA repair, replication fork stability, and prevention of parthanatos. NAD^+^ levels decline with age, correlating with mitochondrial dysfunction, DNA damage accumulation, impaired autophagy and mitophagy, increased ROS, and neuroinflammation [157]. Maintaining adequate NAD^+^ levels is essential for brain function, mitochondrial protection, DNA repair, and resistance to oxidative stress [156].

To modulate NAD^+^ metabolism, several therapeutic strategies have been developed, including supplementation with NAD^+^ and its precursors, inhibition of NAD^+^-consuming enzymes, and activation of NAD^+^ biosynthetic pathways [158]. The most commonly used precursors include nicotinamide (NAM), nicotinamide mononucleotide (NMN), and nicotinamide riboside (NR). Oral NR administration elevates NAD^+^ concentrations in humans (up to ~2.7-fold) and mice, with well-defined pharmacokinetics [158,159,160], and raises hepatic NAD^+^ more efficiently than nicotinic acid or NAM [160]. In healthy volunteers, NR increases circulating NAD^+^ without significant adverse effects, supporting its safety and translational potential [161,162,163]. Preclinical studies show that NAD^+^ precursors exert anti-inflammatory, antioxidant, and neuroprotective effects under ischemic and hypoxic conditions [161]. In neurodegenerative models (3xTgAD/Polβ+/− mice), NR reduces DNA damage, neuroinflammation, and apoptosis, while enhancing SIRT3 activity and improving cognition and synaptic plasticity [164]. Notably, NR may provide stronger neuroprotection than direct NAD^+^ administration under NMDA-induced excitotoxicity, likely due to better maintenance of intracellular NAD^+^ homeostasis [165].

Similar effects have been reported for NMN. Post-ischemic NMN administration exerts neuroprotective actions, preserving CA1 hippocampal neurons by reducing poly(ADP-ribose) formation and inhibiting excessive PARP1-dependent NAD^+^ consumption [159]. Thus, NAD^+^ replenishment counteracts PARP1-induced energetic collapse [166]. NMN may also enhance SIRT1 activity, reduce oxidative stress, and improve vascular function in aging models [167]. Due to the limited bioavailability of classical NAD^+^ precursors (NR, NMN), novel compounds with improved stability and efficacy are being explored [157]. Reduced precursor forms, such as NRH and NMNH, have been shown to increase NAD^+^ levels more effectively both in vitro and in vivo [168,169]. Another promising compound is trigonelline, which demonstrates beneficial effects in models of aging and cognitive impairment [170,171,172].

An alternative strategy to increase NAD^+^ levels involves inhibiting NAD^+^-consuming enzymes such as CD38 and PARP1 [158]. Natural compounds, including cyanidin-3-O-glucoside, apigenin, and quercetin, inhibit CD38 activity, increase the NAD^+^/NADH ratio, and improve mitochondrial function [173,174,175]. Apigenin, in particular, elevates NAD^+^ levels, improves metabolic homeostasis, activates SIRT3, and restores mitochondrial function [176,177]. The anti-CD38 antibody TNB-738 represents a novel therapeutic approach for boosting NAD^+^ levels [178]. Inhibition of PARP1 also increases NAD^+^ availability and activates SIRT1 [179]. PARP1 inhibitors such as olaparib, niraparib, and 3,4-dihydro-5-[4-(1-piperidinyl)butoxy]-1(2*H*)-isoquinolinone (DPQ) reduce NAD^+^ consumption by blocking the enzyme’s catalytic activity [180,181,182]. In models of cerebral ischemia, PARP1 inhibition (e.g., with 3-aminobenzamide) reduces lesion size and attenuates disturbances in neurotransmission [183,184].

The NAD^+^ salvage pathway also plays a critical role, with nicotinamide phosphoribosyl transferase (NAMPT) as a key regulatory enzyme. Preclinical studies have shown that enhancement of NAMPT activity can mitigate ischemic brain injury by preserving NAD^+^ availability, activating SIRT1-dependent signaling pathways, promoting axonal remodeling, and improving neurological outcomes in experimental stroke models [185]. While these findings highlight the therapeutic potential of targeting NAMPT, evidence from human studies remains largely observational and insufficient to confirm a causal neuroprotective effect. Accordingly, a phase-specific therapeutic paradigm has been proposed, consisting of early PARP1 inhibition to prevent excessive NAD^+^ consumption, followed by restoration of NAD^+^ pools and support of NAMPT-dependent NAD^+^ biosynthesis during the recovery phase [156]. Further clinical studies are required to determine whether this strategy translates into improved outcomes in patients with ischemic stroke.

Building upon its established role in astrocytic redox regulation, Nrf2 also represents a critical determinant of antioxidant defense under ischemia–reperfusion (I/R) conditions. Nrf2 induces the expression of antioxidant genes, supports mitochondrial function, reduces endoplasmic reticulum stress, attenuates inflammation, decreases blood–brain barrier permeability, and inhibits neuronal apoptosis [186].

Activation of Nrf2 in astrocytes increases the expression of GPx4, GSH synthesis enzymes glutamate-cysteine ligase catalytic subunit (GCLC), glutamate-cysteine ligase modifier subunit (GCLM), xCT, and PPP components, leading to enhanced NADPH production and improved GSH regeneration capacity. In the ischemic penumbra, activation of the Nrf2–GPx4 axis limits lipid peroxidation, reduces oxidative stress, and protects DNA from damage [187].

The Nrf2–GPx4–ferroptosis suppressor protein 1 (FSP1) axis, supported by autophagic processes, represents a promising therapeutic target in the ischemic penumbra, enabling maintenance of astrocytic redox capacity and limiting damage progression [188,189]. Similarly, ischemic preconditioning (IPC) promotes ischemic tolerance by activating Nrf2-dependent adaptive pathways, stabilizing mitochondrial function, and suppressing Nrf2 inhibitors such as GSK3β and Keap1 [190,191,192,193].

A variety of pharmacological and natural Nrf2 activators have demonstrated neuroprotective effects in experimental stroke models. These include electrophilic and multi-target Keap1 inhibitors as well as natural compounds such as 11-keto-β-boswellic acid, 3,14,19-triacetyl andrographolide, icariside II, curcumin, nomilin, acetyl-11-keto-β-boswellic acid, resveratrol, and extracts of *Cistanche deserticola* and *Gastrodia elata* [194,195,196,197,198,199,200,201,202,203,204,205]. These agents have been reported to reduce oxidative stress, attenuate inflammation, preserve blood–brain barrier integrity, and improve neuronal survival in preclinical studies [194,195,196,197,198,199,200,202,203,204,205].

Clinical studies directly evaluating Nrf2-targeted interventions in patients with ischemic stroke are scarce, and no Nrf2 activator has yet demonstrated conclusive efficacy in improving neurological outcomes after I/R brain injury. Furthermore, the acute nature of stroke may limit the effectiveness of therapies that rely on transcriptional activation and de novo protein synthesis, potentially reducing their efficacy within the narrow therapeutic window available after ischemic injury [206,207,208]. Additional concerns relate to the safety of sustained Nrf2 activation. Although transient activation of Nrf2 is generally considered cytoprotective, chronic stimulation may disrupt physiological redox signaling and cellular homeostasis. Persistent Nrf2 activity has been associated with enhanced survival of damaged or transformed cells, promotion of tumor growth, increased metastatic potential, and resistance to chemotherapy and radiotherapy in certain cancer types [209]. Moreover, the clinical development of some potent Nrf2 activators has raised safety concerns. For example, bardoxolone methyl demonstrated promising antioxidant and anti-inflammatory effects but was associated with an increased incidence of cardio-vascular adverse events, including heart failure, leading to premature termination of a large clinical trial in patients with diabetic nephropathy [210]. Additional challenges include the pleiotropic nature of Nrf2 signaling, variability in patient responses, potential interactions with concomitant medications, and the lack of biomarkers to identify patients most likely to benefit from Nrf2-targeted therapies. Therefore, careful optimization of treatment timing, dosage, and duration will be essential to maximize neuroprotection while minimizing potential adverse effects [206,207,208].

Following hypoxia and ischemia/reperfusion (I/R), astrocytes undergo metabolic reprogramming characterized by enhanced glycolysis and activation of the PPP through increased G6PD activity, its rate-limiting enzyme [74,211,212]. As a major source of NADPH, the PPP supports the regeneration of reduced glutathione, thereby strengthening antioxidant defenses and limiting ROS-mediated damage [67]. Increased expression of TP53-induced glycolysis and apoptosis regulator (TIGAR) further promotes PPP flux and suppresses NF-κB signaling, contributing to reduced neuroinflammation in experimental models of cerebral ischemia [213].

Astrocytes also counteract glutamate excitotoxicity by enhancing glutamate uptake, a process associated with increased lactate production and further stimulation of PPP activity [214,215]. Regulation of G6PD occurs at both metabolic and transcriptional levels, with the Keap1/Nrf2 pathway serving as a major regulatory mechanism [74,216].

During reoxygenation, ROS-induced Nrf2 activation promotes PPP activity and GSH synthesis. Accordingly, Nrf2 activators such as sulforaphane increase PPP flux and antioxidant capacity in astrocytes, while dimethyl fumarate exerts similar effects through activation of Nrf2 signaling [40,53,217]. In contrast, some Nrf2 activators, including bardoxolone methyl, have limited neuroprotective potential due to poor blood–brain barrier penetration [218,219,220].

Microglia-derived nitric oxide (NO) can further enhance astrocytic antioxidant defenses by stimulating Nrf2-dependent G6PD expression and PPP activity, thereby increasing GSH availability and indirectly protecting neurons from oxidative injury [221,222]. Collectively, these findings suggest that preservation of G6PD/PPP activity may represent a promising neuroprotective strategy by maintaining astrocytic redox homeostasis during ischemic injury [221,222]. However, the majority of evidence supporting the neuroprotective role of astrocytic PPP activation originates from cell culture and animal studies. Evidence from human studies remains indirect. Clinical and observational studies have demonstrated that oxidative stress markers, glutathione homeostasis, and Nrf2-regulated antioxidant pathways are altered in patients with ischemic stroke and are associated with stroke severity and functional outcome [40,217]. Furthermore, pharmacological Nrf2 activation has been validated in humans for other neurological and inflammatory disorders, most notably with dimethyl fumarate, confirming the feasibility of targeting this pathway in clinical settings [40,217]. However, no clinical trials have specifically evaluated modulation of astrocytic G6PD activity, PPP flux, or TIGAR signaling in patients with ischemic stroke. Consequently, the relevance of these mechanisms to human stroke is currently inferred primarily from biomarker studies and indirect translational evidence rather than direct therapeutic interventions [40,217,223].

Mitochondria play a key role in the pathophysiology of ischemic stroke, as their dysfunction leads to a rapid increase in ROS, disruption of oxidative phosphorylation, loss of mitochondrial membrane potential, and activation of cell death pathways. Impaired bioenergetics, abnormal mitochondrial morphology and structure, and dysregulation of mitochondrial dynamics are critical factors that initiate signaling cascades leading to cell death [224]. Interventions targeting mitochondrial quality control and dynamics, both pharmacological and genetic, have demonstrated neuroprotective effects in preclinical studies [225,226]. Unfortunately, clinical trials using mitochondrial protective agents or antioxidants, whether as monotherapy or in combination therapy, have not yielded the expected results [227].

In recent years, attention has been drawn to astrocytes’ ability to compensate for neuronal damage by transferring functional mitochondria, a novel mechanism of metabolic rescue. The transferred organelles restore ATP production, stabilize Ca^2+^ homeostasis, reduce ROS generation, and support neuronal antioxidant systems [228]. Astrocytes transfer mitochondria to neurons through tunneling nanotubes (TNTs), extracellular vesicles (EVs), Cx43-dependent gap junctions, and direct membrane fusion. Pharmacological enhancement of this process (e.g., via modulation of Miro1, AMPK, or PI3K/AKT signaling) or transplantation of exogenous mitochondria reduces infarct volume and improves neuronal survival in MCAO models [228]. AMPK, PI3K/AKT, and brain-derived neurotrophic factor (BDNF) promote cellular bioenergetics, mitochondrial biogenesis, and mobility, thereby facilitating astrocyte–neuron mitochondrial transfer [229,230]. Mitochondrial mobility depends on transport proteins and Cx43-mediated gap junctions [231,232,233]. In contrast, excessive Drp1 activity induces pathological mitochondrial fragmentation after stroke, impairing mitochondrial transfer and regenerative capacity; therefore, its inhibition exerts neuroprotective effects [234].

Modern delivery systems improve both mitochondrial survival and the precision of targeting to damaged neurons. Transplanted mitochondria can integrate with host cells, contributing to improved energy metabolism, restoration of mitochondrial function, and prevention of cell death [235,236,237]. Studies in animal models have demonstrated improvements in bioenergetic function, reduction in ROS, attenuation of inflammation, and protection against apoptosis [235,238,239]. Preliminary clinical studies suggest that autologous mitochondrial transplantation may improve organ function after ischemia–reperfusion injury without significant adverse effects [240,241], and the first clinical trial in stroke (NCT04998357) is currently underway [235,242].

Despite promising preclinical findings, the clinical translation of mitochondrial transfer and transplantation remains challenging. Most evidence regarding astrocyte-to-neuron mitochondrial transfer, Miro1, AMPK, PI3K/AKT signaling, and Drp1 inhibition comes from in vitro and animal studies of ischemic stroke [228,229,230,231,232,233,234]. To date, no mitochondria-targeted therapy has demonstrated efficacy in randomized clinical trials. Clinical experience with mitochondrial transplantation is limited mainly to cardiac ischemia–reperfusion injury, where early studies have shown feasibility and safety [240,241]. In ischemic stroke, evidence is currently restricted to an ongoing early-phase clinical trial (NCT04998357) [235,242]. Major barriers include limited mitochondrial viability, lack of standardized protocols, delivery challenges, restricted blood–brain barrier penetration, and insufficient knowledge of long-term integration. Therefore, the clinical efficacy of these approaches in ischemic stroke remains to be established [235,242].

One-carbon (1C) metabolism integrates nutrient availability with redox regulation, epigenetic processes, and cellular biosynthesis. Deficiencies of folate, vitamin B6, vitamin B12, methionine, choline, or disturbances in transsulfuration contribute to ischemic stroke risk and progression [243,244,245,246,247,248,249,250,251].

Experimental studies indicate that supplementation with B vitamins, folate, and choline enhances neuroplasticity, antioxidant defenses, and post-stroke recovery, whereas methylenetetrahydrofolate reductase deficiency exacerbates neuronal injury [252,253].

Choline and its derivative citicoline have demonstrated neuroprotective effects in experimental stroke models through membrane repair, reduction in oxidative stress, and promotion of functional recovery [254,255,256]. In addition, methionine metabolism and the transsulfuration pathway may contribute to neuroprotection by regulating NF-κB signaling and glutathione synthesis, although these mechanisms require further investigation [257,258,259].

Despite promising preclinical findings, clinical evidence remains inconsistent [248]. Several studies have shown that supplementation with folic acid, vitamin B6, and vitamin B12 lowers homocysteine levels but does not consistently improve cognitive outcomes or reduce recurrent vascular events after stroke [260,261].

However, folic acid supplementation has been associated with reduced risk of first stroke in selected populations, particularly in regions without mandatory folic acid fortification and among hypertensive individuals [262,263,264,265,266,267,268].

Overall, current clinical data support a role for 1C metabolism primarily in stroke prevention rather than post-stroke neurorestoration. While homocysteine lowering is biologically plausible and supported by epidemiological evidence, the beneficial effects of folate, B vitamins, choline, citicoline, or transsulfuration-targeted interventions observed in experimental models have not yet been consistently translated into improved clinical outcomes in patients with ischemic stroke [248,260,261,262,263,264,265,266,267,268].

Preservation of metabolic coupling between astrocytes and neurons is considered a promising strategy for maintaining redox homeostasis and limiting injury progression within the ischemic penumbra. Owing to their high glycolytic activity and ability to generate NADPH through the pentose phosphate pathway, astrocytes play a central role in buffering oxidative stress and sustaining glutathione-dependent antioxidant defenses [269].

Through the astrocyte–neuron lactate shuttle, astrocytes export lactate via MCT1 and MCT4, whereas neurons take it up through MCT2, supporting mitochondrial metabolism and reducing oxidative stress [270,271,272]. Disruption of this coupling contributes to energetic failure, NAD^+^ depletion, ROS accumulation, and secondary cellular damage. Consequently, strategies aimed at preserving lactate transport, astrocytic glycolysis, and neuronal lactate utilization have been proposed as potential neuroprotective approaches [270,271,272,273,274,275,276,277].

Experimental studies have demonstrated that exogenous lactate administration reduces infarct volume, attenuates excitotoxicity, and improves metabolic recovery following cerebral ischemia [270,278,279,280,281,282,283,284]. In addition, lactate-dependent signaling through HCAR1 and regulation of monocarboxylate transporters by BDNF, HIF-1α, and AMPK may further influence neuronal resilience and post-ischemic recovery [273,274,275,276,277,280,281,282,283,284,285,286,287,288,289,290].

Although lactate administration improves brain energy metabolism in trauma patients [270,279], evidence that modifying the astrocytic-neuronal axis (ANLS), MCTs, or HCAR1 signaling improves clinical outcomes after ischemic stroke is still limited. Therefore, the neuroprotective effects of lactate demonstrated in experimental models have not yet been unequivocally confirmed in humans [273,274,275,276,277,280,281,282,283,284,285,288,289,290].

Modulation of microglia–astrocyte interactions represents a promising strategy for protecting the ischemic penumbra. Excessive activation of M1 microglia promotes the release of iNOS-derived nitric oxide (NO), TNF-α, IL-1β, IL-6, and C1q, driving the formation of neurotoxic A1 astrocytes and amplifying oxidative and nitrosative stress [291,292,293,294,295,296].

Elevated NO and peroxynitrite levels induce DNA damage, activate PARP1, deplete NAD^+^, and impair astrocytic antioxidant capacity, thereby contributing to secondary neuronal injury [291,292,293,294,295,296,297].

Several selective iNOS inhibitors, including N-(3-(aminomethyl)benzyl)acetamidine, L-N6-(1-iminoethyl)-lysine (L-NIL), GW274150, and 2-iminobiotin, have demonstrated neuroprotective effects in experimental models by reducing NO production and nitrosative damage [161,162,163,297,298]. Pharmacological inhibition of the TNF-α/IL-1α/C1q signaling axis, including clinically approved agents such as etanercept, infliximab, adalimumab, golimumab, anakinra, rilonacept, and canakinumab, reduces neuroinflammation and tissue injury in experimental stroke models [299,300,301,302,303,304,305,306]. Similarly, C1q neutralization and inhibition of inflammatory transcription factors such as STAT1 and NF-κB attenuate microglia-mediated neurotoxicity [307,308].

Cell-based therapies, particularly mesenchymal stem cells (MSCs), MSC-derived extracellular vesicles (MSC-EVs), and neural stem/precursor cells (NSCs/NPCs), consistently promote a shift from proinflammatory M1 microglia toward reparative M2 phenotypes, reducing neuroinflammation and supporting tissue repair in experimental stroke models [309,310,311,312,313,314,315,316,317,318,319,320,321,322,323,324,325,326,327,328,329,330,331,332]. NPCs additionally suppress proinflammatory mediators (IL-1β, IL-6, TNF-α, iNOS) and increase reparative cell populations, including CD206^+^, Arg1^+^, and IGF1^+^ cells [325,333,334,335,336,337,338,339,340,341,342,343,344,345,346,347,348,349].

More recently, the IL-3/IL-3Rα and VEGFD/VEGFR3 signaling pathways have been identified as regulators of microglial lipid metabolism and inflammatory activation. Experimental studies suggest that restoring IL-3 signaling or inhibiting VEGFR3 improves mitochondrial β-oxidation, enhances phagocytic activity, reduces inflammation, and promotes recovery after ischemia–reperfusion injury [295,309,312,313,314,315,316,317,318,319,320,321,322,323,324,325,326,327,328,329,330,331,332].

Despite promising preclinical results, the clinical translation of therapies modulating microglia-astrocyte signaling in ischemic stroke remains unproven. Anti-inflammatory drugs (anakinra, TNF-α inhibitors) and MSCs have not yet demonstrated a clear improvement in functional outcomes, while the potential of MSC-EVs, NPCs, and the IL-3/IL-3Rα and VEGFD/VEGFR3 axes has been studied primarily in vitro and in animals. This strategy needs to be verified in well-designed, randomized clinical trials [299,300,301,302,303,304,305,306,307,308,309,310,311,312,313,314,315,316,317,318,319,320,321,322,323,324,325,326,327,328,329,330,331,332,333,334,335,336,337,338,339,340,341,342,343,344,345,346,347,348,349].

In recent years, increasing attention has been paid to the role of senescent cells in the pathophysiology of the ischemic penumbra. Astrocyte senescence leads to the development of the SASP (senescence-associated secretory phenotype), a proinflammatory and pro-oxidative state that exacerbates oxidative stress, destabilizes redox homeostasis, and increases neuronal susceptibility to DNA damage. Therefore, therapeutic strategies targeting the elimination of senescent cells (senolytics) or modulation of SASP (senomorphics) are emerging as promising approaches for protecting the ischemic penumbra. Rather than directly targeting neurons, these interventions focus on preserving astrocytic redox capacity, which is critical for maintaining DNA integrity and limiting secondary tissue damage after ischemia. Senolytics such as dasatinib and quercetin, as well as newer generations of inhibitors targeting pro-survival pathways in senescent cells, have demonstrated the ability to reduce SASP burden and improve neural tissue function in models of aging and neurodegeneration [350].

Senomorphics, including mechanistic target of rapamycin inhibitors, janus kinase (JAK) inhibitors, and epigenetic modulators, can suppress SASP without eliminating cells, thereby reducing the risk of adverse effects and potentially making them more suitable for acute conditions such as ischemic stroke [351]. Modulation of SASP improves synaptic plasticity, reduces neuroinflammation, and supports cognitive function, highlighting the therapeutic potential of these approaches in the ischemic penumbra [352].

Senotherapies, both senolytic and senomorphic, may become a key component of penumbra-protective strategies by shifting the focus from neuron-centered interventions toward control of astrocytic oxidative stress and SASP, which ultimately determine neuronal survival in vulnerable regions [350].

Although senolytic and senomorphic strategies reduce oxidative stress and secondary brain damage by targeting astrocyte senescence and SASP, their clinical utility remains uncertain and is primarily based on experimental models [350,351,352]. Due to cellular heterogeneity, transient senescence in the acute phase may promote tissue remodeling, and its complete elimination may therefore compromise repair processes. Other limitations include the lack of patient selection markers and the risks of toxicity (hematological and cardiovascular with dasatinib) and infection (with rapamycin and JAK inhibitors). Clinical translation of these approaches requires validation in randomized trials [350,351,352].

Based on current evidence, activation of endogenous antioxidant pathways, particularly Nrf2 signaling, together with NAD^+^-preserving therapies and cell-based approaches employing mesenchymal stem cells (MSCs) and their extracellular vesicles, represent the most advanced translational strategies for protecting the ischemic penumbra (Table 1) [40,154,156,157,161,162,163,164,165,166,167,168,169,170,171,172,173,174,175,176,177,178,179,180,181,182,183,184,185,186,187,188,189,190,191,192,193,194,195,196,197,198,199,200,201,202,203,204,205,206,207,208,209,210,217,309,310,311,312,313,314,315,316,317,318,319,320,321,322,323,324,325,326,327,328,329,330,331,332]. At the same time, several emerging approaches, including mitochondrial transplantation, senotherapeutics, and metabolic reprogramming of glial cells, remain at an early stage of development despite encouraging preclinical results [224,225,226,227,228,229,230,231,232,233,234,235,236,237,238,239,240,241,242,291,292,293,294,295,296,297,298,299,300,301,302,303,304,305,306,307,308,309,310,311,312,313,314,315,316,317,318,319,320,321,322,323,324,325,326,327,328,329,330,331,332,333,334,335,336,340,341,342,343,344,345,346,347,348,349,350,351,352].

Taken together, current evidence highlights the therapeutic potential of targeting oxidative stress, metabolic dysfunction, and DNA damage in cerebral ischemia, although successful clinical translation remains limited. Many interventions that have shown robust efficacy in experimental models have failed to demonstrate clear clinical benefit in patients. Major challenges include the narrow therapeutic window of acute ischemic stroke, patient heterogeneity, comorbidities, and the inherent differences between experimental models and clinical reality. At present, restoration of cerebral blood flow through reperfusion therapy remains the only proven strategy for salvaging penumbral tissue. Future therapeutic advances will likely depend on combining reperfusion therapies with interventions targeting oxidative stress, metabolic failure, mitochondrial dysfunction, and DNA damage in a time-dependent and patient-specific manner. However, rigorous translational research and well-designed randomized clinical trials are still required to determine whether these promising experimental approaches can provide meaningful clinical benefit in ischemic stroke [135,353].

## 7. Conclusions

Ischemic stroke leads to a profound destabilization of redox balance and metabolism within the penumbra, where neurons remain viable yet operate on the brink of energetic collapse. Excessive production of ROS and RNS, arising from mitochondrial dysfunction, NADPH oxidase activation, and ionic disturbances, generates a wide spectrum of DNA lesions, ranging from base modifications and AP sites to single- and double-strand breaks. The fate of neurons is determined not only by the extent of this damage but also by their capacity for repair, which becomes severely compromised under conditions of NAD^+^ and ATP depletion. Excessive PARP1 activation, which consumes NAD^+^, further deepens the bioenergetic crisis and accelerates cell death.

Within this critical environment, astrocytes serve as the principal stabilizers of redox homeostasis and metabolism. Their high pentose phosphate pathway activity, ability to generate NADPH, and maintenance of glutathione pools make them the brain’s primary antioxidant buffer. Astrocytes support neurons by supplying lactate, regulating mitochondrial function, maintaining ionic balance, and clearing glutamate, thereby protecting against excitotoxicity. NRF2 activation enhances their antioxidant capacity and boosts GSH synthesis, while peroxisomal and mitochondrial detoxification mechanisms provide additional protection against ROS. Astrocytes also modulate inflammatory responses, shaping microglial phenotypes and limiting the production of toxic mediators.

Astrocytes do not protect neuronal genomes by directly regulating DNA repair pathways but rather by maintaining the metabolic environment required for their effective function. Stabilization of NAD^+^, mitochondrial support, control of the astrocyte–neuron lactate shuttle, and buffering of ROS and RNS, together with glutamate regulation, form an integrated redox–metabolic axis that determines the reversibility of the penumbra. Understanding these mechanisms opens the way to therapeutic strategies aimed at preserving astrocytic metabolic capacity, including NAD^+^ augmentation and modulation of NRF2 and PARP1, as well as mitochondrial-targeted interventions, which may improve neuronal survival and clinical outcomes after stroke.

However, despite strong mechanistic rationale and encouraging preclinical findings, the clinical translation of these approaches remains limited. Therefore, their therapeutic potential in ischemic stroke requires validation in rigorous translational studies and well-designed randomized clinical trials.

## Figures and Tables

**Table 1:** Translational prioritization of candidate therapeutic strategies for ischemic stroke based on mechanistic rationale and current clinical evidence.

Therapeutic Strategy	Strength of Preclinical Evidence	Clinical Evidence	Translational Potential	References
Nrf2 activation and antioxidant response modulation	Very strong	Indirect clinical evidence (e.g., dimethyl fumarate, oxidative stress biomarkers)	High	[186,187,188,189,190,191,192,193,194,195,196,197,198,199,200,201,202,203,204,205,206,207,208,209,210] [40,217]
NAD^+^ preservation and replenishment (NR, NMN, NAMPT activation, PARP1/CD38 inhibition)	Very strong	Human studies demonstrate safety and NAD^+^ elevation, but no proven efficacy in ischemic stroke	High	[154,156,157,161,162,163,164,165,166,167,168,169,170,171,172,173,174,175,176,177,178,179,180,181,182,183,184,185]
Mesenchymal stem cells (MSCs) and MSC-derived extracellular vesicles (MSC-EVs)	Very strong	Early-phase clinical trials demonstrating safety and feasibility	High	[309,310,311,312,313,314,315,316,317,318,319,320,321,322,323,324,325,326,327,328,329,330,331,332]
One-carbon metabolism modulation (folate, vitamins B6/B12, homocysteine lowering)	Strong	Multiple clinical studies and meta-analyses, particularly in stroke prevention	Moderately high	[243,244,245,246,247,248,249,250,251,252,253,260,261,266,267] [262,263,264,265,268]
Preservation of astrocyte–neuron metabolic coupling and lactate shuttle (ANLS)	Strong	Limited human evidence; metabolic effects demonstrated in brain injury patients	Moderate	[269,270,271,272,273,274,275,276,277,278,279,280,281,282,283,284,285,286,287,288,289,290]
Mitochondrial quality control and mitochondrial dynamics	Very strong	Largely unsuccessful clinical translation to date	Moderate	[224,225,226,227]
Mitochondrial transfer and mitochondrial transplantation	Very strong	Early clinical experience, ongoing stroke trials only	Moderate–low	[228,229,230,231,232,233,234,235,236,237,238,239,240,241,242]
Microglia–astrocyte interaction modulation (TNF-α, IL-1, C1q, iNOS pathways)	Strong	Limited clinical evidence in stroke; some agents are approved for other inflammatory diseases	Moderate–low	[291,292,293,294,295,296,297,298,299,300,301,302,303,304,305,306,307,308] [309,310,311,312,313,314,315,316,317,318,319,320,321,322,323,324,325,326,327,328,329,330,331,332,333,334,335,336,340,341,342,343,344,345,346,347,348,349]
Senotherapeutic approaches (senolytics and senomorphics)	Promising	Very limited clinical evidence; none specifically established in stroke	Low	[350,351,352]
IL-3/IL-3Rα and VEGFD/VEGFR3 signaling modulation	Preliminary	No clinical stroke studies available	Very low	[295]

Abbreviations: ANLS—astrocyte–neuron lactate shuttle; B6—vitamin B6 (pyridoxine); B12—vitamin B12 (cobalamin); C1q—complement component 1q; CD38—cluster of differentiation 38; EVs—extracellular vesicles; IL-1—interleukin-1; IL-3—interleukin-3; IL-3Rα—interleukin-3 receptor alpha; iNOS—inducible nitric oxide synthase; MSCs—mesenchymal stem cells; NAD^+^—nicotinamide adenine dinucleotide (oxidized form); NAMPT—nicotinamide phosphoribosyltransferas; NMN—nicotinamide mononucleotide; NR—nicotinamide riboside; Nrf2—nuclear factor erythroid 2–related factor 2; PARP1—poly(ADP-ribose) polymerase 1; TNF-α—tumor necrosis factor alpha; VEGFD—vascular endothelial growth factor D; VEGFR3—vascular endothelial growth factor receptor 3.

## Data Availability

No new data were created or analyzed in this study.

## Abbreviations

The following abbreviations are used in this manuscript:

ADSC	adipose-derived stem cell
AIF	apoptosis-inducing factor
AKT	protein kinase B
AMPK	AMP-activated protein kinase
ANLS	astrocyte–neuron lactate shuttle
APE1	apurinic/apyrimidinic endonuclease 1
AP	apurinic/apyrimidinic site
ARE	antioxidant response element
ATP	adenosine triphosphate
B6	vitamin B6 (pyridoxine)
B12	vitamin B12 (cobalamin)
BDNF	brain-derived neurotrophic factor
BER	base excision repair
BH4	tetrahydrobiopterin
BMSC	bone marrow mesenchymal stem cell
CaMKII	calcium/calmodulin-dependent protein kinase II
cAMP	cyclic adenosine monophosphate
CBS	cystathionine beta-synthase
CD38	cluster of differentiation 38
C1q	complement component 1q
CI/R	cerebral ischemia/reperfusion
CNS	central nervous system
CPT1A	carnitine palmitoyltransferase 1A
Cx43	connexin 43
CXCL10	C-X-C motif chemokine ligand 10
CXCR4	C-X-C motif chemokine receptor 4
DDR	DNA damage response
DNA	deoxyribonucleic acid
DPQ	PARP inhibitor
Drp1	dynamin-related protein 1
DSB/DSBs	double-strand break(s)
eNOS	endothelial nitric oxide synthase
ERCC1	excision repair cross-complementation group 1
EVs	extracellular vesicles
FSP1	ferroptosis suppressor protein 1
GABA	gamma-aminobutyric acid
G6PD	glucose-6-phosphate dehydrogenase
GCLC	glutamate-cysteine ligase catalytic subunit
GCLM	glutamate-cysteine ligase modifier subunit
Gh	guanidinohydantoin
GSH	glutathione
GSK3β	glycogen synthase kinase 3 beta
GLUT1	glucose transporter type 1
GPx	glutathione peroxidase
H2AX	H2A histone family member X
HCAR1	hydroxycarboxylic acid receptor 1
HGF	hepatocyte growth factor
HIF-1α	hypoxia-inducible factor 1-alpha
HMGB1	high mobility group box 1
HLL	NF-κB reporter system
HO-1	heme oxygenase-1
hUCMSC	human umbilical cord mesenchymal stem cells
IL-1β	interleukin 1 beta
IL-3–IL-3Rα	interleukin 3 and receptor
IL-6	interleukin 6
IL-12β	interleukin 12 beta
iNOS	inducible nitric oxide synthase
IPC	ischemic preconditioning
I/R	ischemia–reperfusion
JAK	Janus kinase
Keap1	Kelch-like ECH-associated protein 1
LDH	lactate dehydrogenase
L-NIL	iNOS inhibitor
LPS	lipopolysaccharide
MAS	malate–aspartate shuttle
MCAO	middle cerebral artery occlusion
MCTs	monocarboxylate transporters
Miro1	mitochondrial Rho GTPase 1
MSC	mesenchymal stem cell
MSC-EVs	MSC-derived extracellular Vesicles
MsrA	methionine sulfoxide reductase A
MTHFR	methylenetetrahydrofolate reductase
mTORC1	mechanistic target of rapamycin complex 1
mTOR	mechanistic target of rapamycin
NAD^+^	nicotinamide adenine dinucleotide
NAM	nicotinamide
NAMPT	nicotinamide phosphoribosyltransferase
NCX	Na^+^/Ca^2+^ exchanger
NEIL	Nei-like DNA glycosylase
NEIL3	Nei-like DNA glycosylase 3
NER	nucleotide excision repair
NF-κB	nuclear factor kappa-light-chain-enhancer of activated B cells
NHEJ	non-homologous end joining
NMDA	N-methyl-D-aspartate (receptor)
NMN	nicotinamide mononucleotide
NMNH	reduced nicotinamide mononucleotide
nNOS	neuronal nitric oxide synthase
NO	nitric oxide
NOX/NOX2	NADPH oxidase family/NADPH oxidase 2
NOS	nitric oxide synthase
Notch	Notch signaling pathway
NR	nicotinamide riboside
NRH	reduced nicotinamide riboside
Nrf2	nuclear factor erythroid 2–related factor 2
NSC	neural stem cell
NPC	neural precursor cell
OGG1	8-oxoguanine DNA glycosylase 1
ONOO^−^	peroxynitrite
8-oxoG	8-oxoguanine
PAR	poly(ADP-ribose)
PARP1	poly(ADP-ribose) polymerase 1
PD	Parkinson’s disease
PDH	pyruvate dehydrogenase complex
PDK1	pyruvate dehydrogenase kinase 1
PDK4	pyruvate dehydrogenase kinase 4
PFKFB3	6-phosphofructo-2-kinase/fructose-2,6-biphosphatase 3
PHDs	prolyl hydroxylase domain enzymes
PI3K	phosphoinositide 3-kinase
PPP	pentose phosphate pathway
RET	reverse electron transport
RNS	reactive nitrogen species
ROS	reactive oxygen species
SAH	S-adenosylhomocysteine
SAM	S-adenosylmethionine
SASP	senescence-associated secretory phenotype
SCI	spinal cord injury
SIRT1/3/6	sirtuins
SOD	superoxide dismutase
SN1/2	sodium-coupled neutral amino acid transporter 1/2
SNAT	sodium-coupled neutral amino acid transporter
Sp	spiroiminodihydantoin
SSB	single-strand break
STC1	stanniocalcin 1
TACE	TNF-α converting enzyme
TBI	traumatic brain injury
TCA	tricarboxylic acid cycle
TET1	ten–eleven translocation 1
TIGAR	TP53-induced glycolysis and apoptosis regulator
TIC	tumor-initiating cells
TLR4	toll-like receptor 4
TNF-α	tumor necrosis factor alpha
TNFR1	tumor necrosis factor receptor 1
TNTs	tunneling nanotubes
TSG-6	TNF-stimulated gene 6
VEGFD/VEGFR3	vascular endothelial growth factor D/receptor
WMI	white matter injury
xCT	cystine/glutamate antiporter
XRCC1	X-ray repair cross-complementing protein 1
1C	one-carbon metabolism
